# Combination of Stable Isotopes and Fatty Acid Composition for Geographical Origin Discrimination of One Argan Oil Vintage

**DOI:** 10.3390/foods10061274

**Published:** 2021-06-03

**Authors:** Sara Elgadi, Ahmed Ouhammou, Fouad Taous, Hamza Zine, Eleni G. Papazoglou, Tibari Elghali, Noureddine Amenzou, Hassan El Allali, Abderrahmane Aitlhaj, Abderraouf El Antari

**Affiliations:** 1 Laboratory of Microbial Biotechnology, Agrosciences and Environment, Faculty of Sciences-Semlalia, Cadi Ayyad University, Marrakech 40000, Morocco; ouhammou@uca.ac.ma (A.O.); hamza.zine@edu.uca.ac.ma (H.Z.); 2 Centre National De L’Energie, des Sciences et Techniques Nucleaires, Rabat 10001, Morocco; taous@cnesten.org.ma (F.T.); elghali@cnesten.org.ma (T.E.); amenzou@cnesten.org.ma (N.A.); 3 Laboratory of Systematic Botany, Department of Crop Science, Agricultural University of Athens, 11855 Athens, Greece; elpapazo@aua.gr; 4 The Interprofessional Federation of the Argan Sector, Agadir 80000, Morocco; elallhas@gmail.com; 5 National Agency for the Development of the Oasis and Argan Zones, Agadir 80000, Morocco; aitlhaj.abderrahmane@gmail.com; 6 Laboratory of Agro, Food Technology and Quality, Regional Center for Agronomic Research of Marrakech, National Institute of Agronomic Research (INRA), Marrakech 40000, Morocco; a_elantari@yahoo.fr

**Keywords:** argan oil, traceability, isotope and elemental techniques, fatty acids, environmental conditions, Morocco

## Abstract

Quality control and traceability of Argan oil requires precise chemical characterization considering different provenances. The fatty acid profile is an essential parameter that certifies the quality and purity of Argan oil. In addition, stable isotopes were recently shown to be accurate as an indicator for geographical origin. In this study, fatty acid composition by gas chromatography (GC) and stable isotope ratio by isotope ratio mass spectrometry (IRMS) were investigated for classifying Argan oil according to its geographical origin. Forty-one Argan oil samples, belonging to six geographical origins of Moroccan natural Argan population (Safi, Essaouira, Agadir Ida Outanane, Taroudant, Tiznit and Sidi Ifni) were collected and extracted under the same conditions. The results show that the isotope δ^13^C, palmitic acid (C16:0), linoleic acid (C18:2) and unsaturated fatty acids (UFA) were strongly influenced by ecological parameters. Linear discriminant analysis (LDA) was performed to discriminate the six studied provenances. Discriminant models predicted the origin of Argan oil with 92.70% success. Samples from Safi, Essaouira and Agadir Ida Outanane presented the highest classification rate (100%). In contrast, the lowest rate was reported for samples from Tiznit (85.70%). The findings obtained for fatty acids and isotope combination might be considered as an accurate tool for determining the geographical origins of Argan oil. Moreover, they can potentially be used as specific markers for oils labeled with Protected Geographical Indication (PGI).

## 1. Introduction

The Argan forest covers an area of 830,000 ha, occurring on the coastal and subcoastal areas of central-western Morocco (the fertile Souss Valley, Anti-Atlas and the coastal regions between Safi and Sidi Ifni) [1]. It was recognized in 1998 as a UNESCO biosphere reserve (Man and the Biosphere Reserve) [2]. This area presents a unique flora composed mainly of Mediterranean taxa, coexisting with tropical, Saharan, Macaronesian and endemic taxa [3,4]. Argan forests provide multiple services, such as carbon sequestration, species habitats, conservation of genetic diversity, a final barrier against desertification [1], prevention of soil erosion, and tourism [5]. The income generated from the Arganeraie Biosphere Reserve e.g., oil from the fruit of Argania spinosa, represents the principal source for many rural households [6]. 

Argan oil is rich in antioxidant compounds such as saponins and tocopherols [7], fatty acids [8], and sterols [9]. It has high nutritional characteristics and relevant medical properties that makes it valuable both in nutrition and cosmetic applications [10]. The variability of Argan oil composition can be attributed to many factors such as climate [11], fruit forms [12], fruit maturity [13], oil extraction method [14], and storage conditions [15]. Nevertheless, few studies have focused on the geographical origin and chemical composition relationship [16]. 

The global Argan oil market is estimated at more than 100 million USD (ANDZOA, 2021 personal communication) and is expected to grow at a revenue-based compound annual growth rate of 10.8% between 2020 and 2027 [17]. Such development requires more control to protect the consumer and the producer from fraud. In fact, several marketing and promotional strategies are aimed to relate food products to their geographical origin. European Union legislation, for example, allows the reservation of geographical designations for food products, such as Protected Designations of Origin (PDO) and Protected Geographical Indications (PGI) [18]. The identification of geographical origin increases consumer confidence in the quality of products. A growing demand was revealed for products that are correctly labeled and related to their geographical origin as local products [18]. 

Under the auspices of the Mohammed VI Foundation for the research and protection of the Argan tree, the Moroccan authorities have set up an internal mechanism for the protection of Argan forests. The aim was the constitution of a legal framework that protects the original products of the Argan tree and links the quality concept to the origin and the geographical indication. On 25 January 2010, Protected Geographical Indication was acquired, enabling the designation ‘Argan Oil’, prepared according to well-defined methods [19], to be given exclusively to products from Central-West Morocco. 

Many approaches have been used for the identification of geographical origin, such as fatty acid composition [20] and Fourier transform infrared fingerprinting [11] combined with chemometric tools as linear discriminant analysis (LDA) and partial least squares–discriminant analysis (PLS-DA) [21]. It was shown by Taous et al. [22] that the pedo-climatic parameters and the isotopic composition of argan oil are linked. The results reported by the same authors showed that it is possible to distinguish the production areas of Argan oil, as well as protect its quality and commercial value [22].

However, few studies have tried to develop models predictive of Argan oil origin based on stable isotope composition [22]. 

Isotopic analysis of the chemical elements of seed extract can help to understand different ecological processes (temperature, precipitation, air humidity) and their influence on the development and physiology of plants during the seed development period, the results generally allow the development of analytical tools to verify the origin of plant material [23]. Miklavčič et al. [20] reported that the fatty acid profile can be used as a tool for confirming the geographical origin of Argan oil. It was recommended as a method for laboratories due to its simplicity, rapidity, and efficiency. In addition, Taous et al. [22] confirmed that stable isotope assessment can provide accurate information on the origin of Argan oil. Consequently, the combination of determining fatty acids and stable isotopes can become a powerful tool for assessing the geographical traceability of Argan oil, in particular, considering its distribution area.

The aim of this study was to characterize the fatty acid profile, and stable isotope composition of Argan oil from six Moroccan provinces: Safi (for the first time), Essaouira, Agadir Ida Outanane, Taroudant, Tiznit and Sidi Ifni to compare the feasibility of combining fatty acid profiling and stable isotope ratio, associated with the chemometric technique LDA, to classify Argan oil according to its geographical origin.

## 2. Materials and Methods

### 2.1. Sampling Area and Plant Material

**Figure 1:**
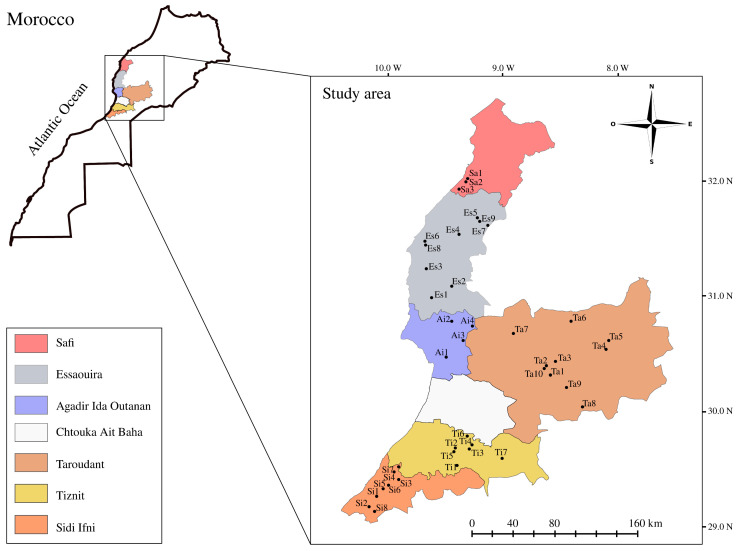
Sampling areas and localization of studied Argan samples.

Argan fruits of natural populations were collected at full maturity from adult trees in six different geographical origins in the Central-West of Morocco from Safi in the North, and Essaouira, Agadir Ida Outanane, Taroudant, Tiznit, and Sidi Ifni in the South (Figure 1). Therefore, the sampled area covered the entire Argan forest. This area is characterized by a semi-arid to arid bioclimate [3]. In addition, the dominant geological ages in the study area are Triassic, Jurassic, and Cretaceous [24]. Geographical parameters of samples were downloaded from Worldclim [25] and included in (Table S1). A total of 41 samples were collected between August and November 2018. After drying, 20 kg of fruit for each study point were depulped and crushed manually between two stones giving between 800 g and 1500 g of kernels for each sample. Kernels obtained were vacuum-sealed until extraction to avoid oxidation.

### 2.2. Oil Extraction

Argan oil was mechanically extracted from unroasted kernels to maintain the natural composition using an oil press (Komet CA59G-IBG Monforts Oekotec). Then, oils were filtered and preserved in dark glass bottles of 250 mL, filled with nitrogen to avoid oxidation, in a refrigerator at +4 °C.

### 2.3. Chemical Composition

#### 2.3.1. Fatty Acids

The determination of fatty acid composition was performed according to the European Union standard methods [26]. Argan oil (1 g) was mixed with 2 mL of petroleum ether and 3 mL of a methanolic potassium hydroxide solution (2 M). Methyl esters were analyzed by gas chromatography (CG, Varian CP 3380) equipped with a capillary column (CP-Wax 52 CB L = 30 m; Φ = 0.25 mm; ø = 0.20μm). The injector temperature was set at 220 °C, and the temperatures of the flame ionization detector (FID) and oven were maintained at 230 °C and 190 °C respectively, with nitrogen used as the carrier gas.

#### 2.3.2. Stable Isotope Analysis

The carbon and nitrogen isotope composition ratios (^13^C/^12^C and ^15^N/^14^N respectively) of Argan oil samples (around 0.3 mg of oil) were determined via continuous flow EA-IRMS using an isotope ratio mass spectrometer (Delta V Thermo Scientific, Germany) coupled to an element analyzer (Thermo Scientific FLASH HT Plus) following the procedure described by Taous et al. [22]. 

The relative difference of isotope ratios (isotope-delta values) of a sample is reported according to the following formula [27]:

δ^i^ E = (^i^ R_SA_ − ^i^ R_REF_)/^i^ R_REF_ (1)

where i is the mass number of the heavier isotope of element E, R_SA_ is the respective isotope ratio of the sample and R_REF_ is the relevant internationally recognized reference material. The delta values were multiplied by 1000 and expressed in units “per mil” (‰).The isotopic δ^15^N‰ and δ^13^C‰ values of the samples were calibrated versus the following certified reference materials: fuel oil NBS-22 (δ^13^C (‰) = −30.031 ± 0.043), benzoic acid IAEA-601(δ^13^C (‰) = −28.81 ± 0.04‰) and Caffeine IAEA-600 (δ^15^N (‰) = +1 ± 0.2‰) (Provided to CNESTEN by IAEA under CRP N° D052040). Uncertainty per batch (7 replicates of secondary isotopic reference material) was ≤1.4‰ for δ^13^C and ≤0.7‰ for δ^15^N.

### 2.4. Statistical Analysis

Statistical analysis was performed with SPSS Statistics version 21 (IBM Corp, Armonk, NY, USA). One-way ANOVA was carried out followed by Tukey as a post-hoc test to determine statistically significant differences between fatty acid and isotopic means for different provinces (*p* < 0.05). Furthermore, linear discriminant analysis (LDA) was applied for building predictive models by the combination of isotopic and fatty acid composition that maximize the discrimination of the predefined regions. The difference between means was normalized by a measure of the within-class variability. In addition, to determine the chemical compounds responsible for determining the geographical origin according to discriminant functions, the statistical significance of each discriminant function was evaluated by Wilk’s lambda. Heatmap and hierarchical clustering were assessed using Ward algorithm and Euclidean distance analysis to determine the relationship between samples according to their geographical parameters using R software version 3.6.2 (R Foundation for Statistical Computing, Vienna, Austria). 

## 3. Results and Discussion

### 3.1. Geographical Parameters

**Figure 2:**
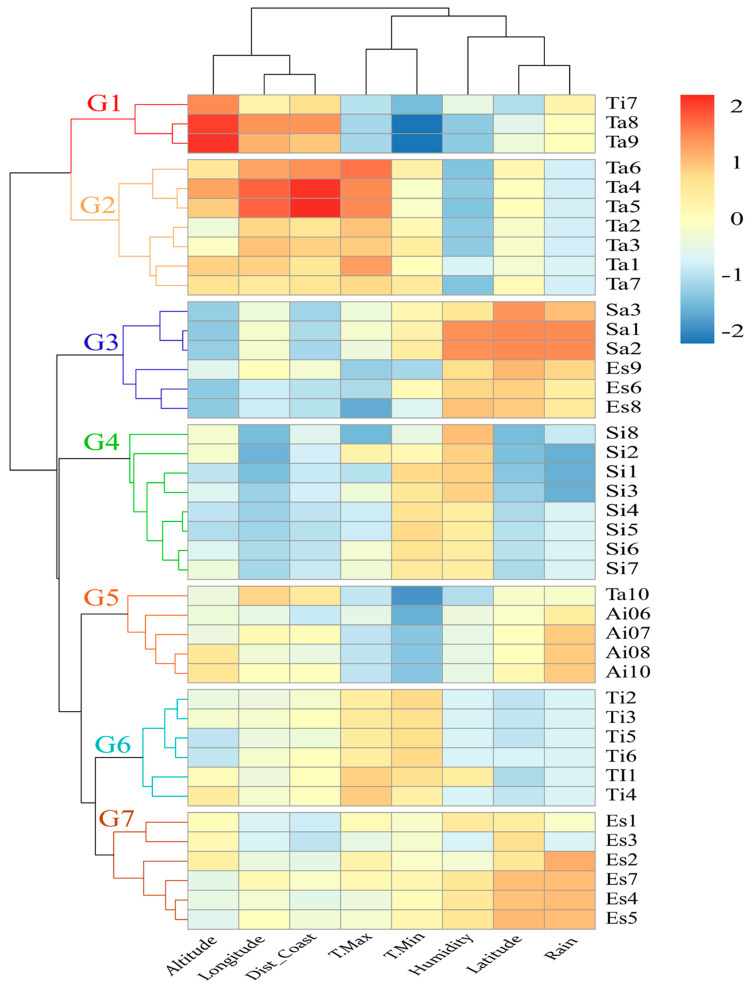
Hierarchical clustering and heatmap of geographical parameters of each sampled location. The rows in the heatmap represent the sampled locations, Safi (Sa), Essaouira (Es), Agadir Ida Outanane (Ai), Taroudant (Ta), Tiznit (Ti) and Sidi Ifni (Si) and the columns indicate geographical parameters, altitude, longitude, distance from the coast (Dist–coast), maximal temperature (T.Max), minimal temperature (T.Min), humidity, latitude and rain. Red and orange cells indicate a higher value for the corresponding geographical parameter and blue cells indicate low values.

The hierarchical clustering analysis of geographical parameters and heatmap (Figure 2) confirmed that each province had specific ecological characteristics with some similarities between adjoining provinces. The first province, Taroudant (G1 and G2), was characterized by high altitude, easternmost longitude and furthest distance from the coast. These groups presented the most continental sampled province. Furthermore, the discrimination between G1 and G2 was also attributed to the differences recorded for both maximal and minimal temperatures. The second group included Safi and Essaouira and was classified based on the high levels of humidity due to their remarkable proximity to the ocean, precipitation and latitude. It represented the coastal sampled locations. The third group, Sidi Ifni, according to high values of minimal temperature and humidity, was considered to be affected by Macaronesian climatic influences. The fourth group, presented by Agadir Ida Outanane, was determined by its medium latitude and precipitation values. The province of Tiznit had medium maximal and minimal temperature values. The last group (6) included the continental sampled locations of Essaouira, which is characterized by a high latitude and precipitation. It should be noted that Ti7 (Tiznit) presents some similarities to Taroudant province (group 1). Furthermore, Ta10 (Taroudant) was classified with those of Agadir Ida Outanane province. The similarities were normal due to the small distances between these provinces. 

### 3.2. Chemical Composition

#### 3.2.1. Fatty Acids

**Figure 3:**
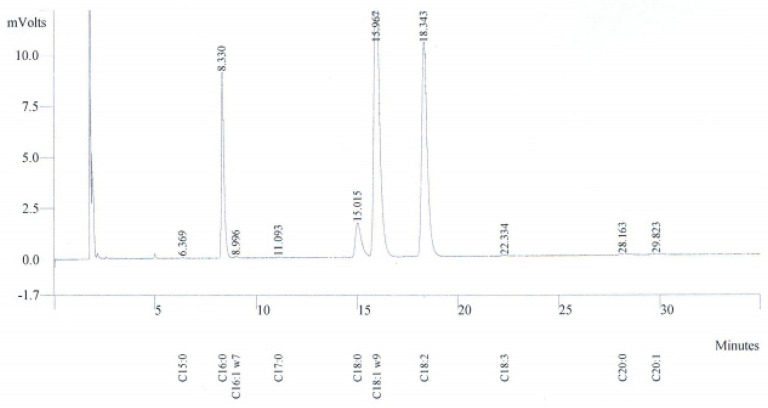
Gas chromatogram (GC–FID) of fatty acid methyl esters of an Argan oil sample.

As shown in (Table 1 and Figure 3), oleic acid was the predominant fatty acid in Argan oil (44.75–48.87%), followed by linoleic acid (29.19–35.23%) and palmitic acid (13.83–14.98%). According to ANOVA followed by Tukey’s test, significant differences between samples from different provinces (*p* < 0.05) were revealed for all studied fatty acids except arachidic acid (C20:0). These results agree with existing studies on unroasted Argan kernels mechanically pressed [16]. The highest oleic acid (C18:1) levels were noted in samples from Agadir Ida Outanane and Taroudant, at 48.87% and 48.80%, respectively. The results of Taroudant are in accordance with findings reported by Kharbach et al. [11] for the same locality. 

Linoleic acid (C18:2) content was high in samples from Safi (35.23%) followed by those from Essaouira (32.84%) and Sidi Ifni (32.12%). These provinces represent the coastal sources of Argan oil. Furthermore, a high percentage of linoleic acid can be used as a marker of coastal Argan oil. Similar results were confirmed by Aithammou et al. [16] and Kharbach et al. [11] for Essaouira. In addition, our results confirmed the high positive correlation also reported by Ait Aabd et al. [10] between linoleic acid and longitude. Samples from Taroudant and Tiznit presented the highest values of palmitic acid, 15.85% and 15.36%, respectively. As previously described, these provinces represented the continental Argan oil, allowing us to consider palmitic acid as a marker of continental Argan oil. Similarly to Kharbach et al. [11] palmitic acid (C16:0) levels increased from the plain (low altitude) to the highlands (high altitude). All values were within the limits established by the SNIMA 08.5.090 standard [28]. Unsaturated fatty acids were eminently high in Argan oils and ranged between 77.98% and 80.45%, while saturated fatty acid contents were lower and varied between 19.42% and 22.03%. 

The results show that the difference of palmitic acid (C16:0), linolenic acid (C18:3), unsaturated fatty acid (UFA) and saturated fatty acid (SFA) between provinces was highly significant (*p* < 0.001) and might be used as markers for the identification geographical origin. 

#### 3.2.2. Isotopic Composition

**Figure 4:**
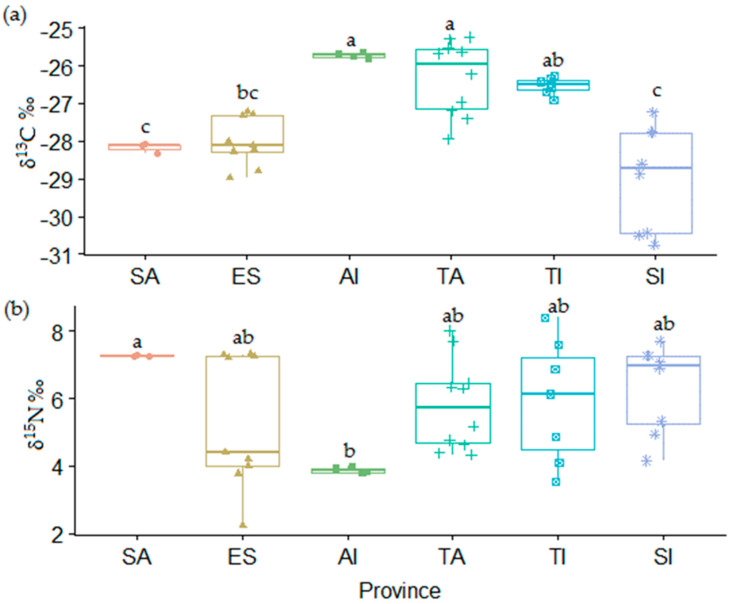
Boxplot of (**a**) δ^13^C and (**b**) δ^15^N in Argan oil samples collected in different provinces (Safi (SA), Agadir Ida Outanane (Ai), Essaouira (ES), Sidi Ifni (SI), Taroudant (TA) and Tiznit (TI). Significant differences (*p* < 0.05) were expressed by different letters.

Stable isotopes ratios δ^13^C and δ^15^N in Argan oil from six regions (provinces) are shown in (Figure 4 and Table S2). δ^13^C values ranged between −30.7‰ and −25.2‰. However, δ^15^N levels were between 0.86‰ and 8.40‰. Taous et al. [22] reported that δ^13^C ratio varied between −29.9‰ and −26.3‰ in Argan oil, which is in agreement with our results. Concerning the δ^15^N ratio, the same authors provided values ranging from +3.1‰ to +7.7‰ for Argan kernels. 

According to ANOVA followed by Tukey’s test, significant differences between samples from different provinces (*p* < 0.05) were highly revealed for δ^13^C. Taroudant, Agadir Ida Outanane and Tiznit presented the highest mean values of δ^13^C, whilst Essaouira, Safi and Sidi Ifni presented much lower means. Similar values were reported for Essaouira (−27.9‰ to −29.9‰) [22]. In sum, high levels of δ^13^C can be used as isotopic marker of continental regions. This finding was supported by the result obtained by Taous et al. [22].

The province of Safi presented the highest level of δ^15^N. In addition, the mean δ^15^N did not differ significantly between Essaouira, Sidi Ifni, Taroudant and Tiznit. The lowest level was shown for samples from Agadir Ida Outanane. According to ANOVA test, δ^15^N in Argan oils may not be appropriate for the identification of geographical origin. The obtained results were consistent with the findings of Taous et al. [22], and with those of Portarena et al. [29] for olive oil.

### 3.3. Relation between Ecological Parameters and Argan Oil Composition 

The results of Pearson correlation analysis (Table 2) showed that δ^13^C, C16:0, C18:2, UFA and SFA were correlated with the majority of geographical parameters. The amount of δ^13^C was affected by longitude, altitude, minimal temperature, humidity and distance from the coast. However, latitude, maximal temperature, and precipitation had no significant relationship with the δ^13^C level. The negative correlation of δ^13^C with humidity might be related to the fact that a high humidity allows stomata to stay open for longer time, facilitating a high passage of CO_2_ inside the leaves and leading to a high accumulation of ^13^C in Argan fruit [30]. δ^15^N had no correlation with geographical parameters. These results are in line with a previous study on stable isotopes [22]. Palmitic acid (C16:0) percentage was positively correlated with the maximal temperature and the distance from the coast. Furthermore, its negative correlation with humidity was revealed. Linoleic acid content (C18:2) was negatively correlated with altitude and the distance from the coast; in contrast, it was positively correlated with humidity. Oleic acid content (C18:1 ω9) was significantly positively correlated (*p* < 0.01) with altitude. Unsaturated fatty acid (UFA) had a positive correlation with latitude; however, a negative correlation was highlighted with longitude and altitude. Ait Aabd et al. [10] reported that Argan oil unsaturated fatty acid content was affected by altitude, latitude and longitude, which our results are consistent with. A similar conclusion was also drawn by Kharbach et al. [31].

### 3.4. Linear Discriminant Analysis (LDA) 

**Figure 5:**
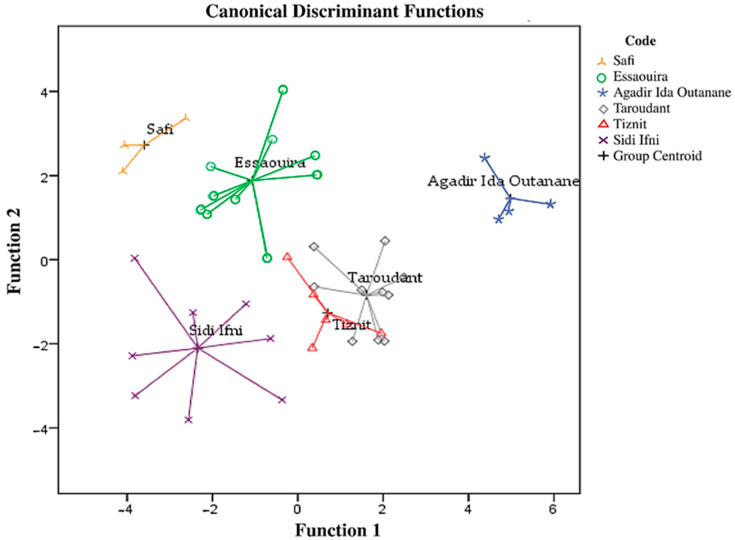
Linear discriminant analysis performed based on a combination of isotopic and fatty acid composition of Argan oil samples from six provinces.

LDA is a supervised method that focuses on maximizing the separability among known categories by creating a new linear axis. In contrast to the PCA, (unsupervised method) groups of objects were predefined. PCA aims to find components that account for maximum variance in the data without taking into account class membership, and is used especially when such information is not available [32]. In our case, the categories (provenances) were known. LDA was performed based on fatty acid and stable isotope combination in order to create discriminant models regarding the geographical origin classification of Argan oils. Figure 4 shows the LDA scatter plot for Argan oils from six provinces. Five discriminant functions were constructed based on Wilks’ lambda values, which explained 100% of the variance (Table S3); 50.94% of the total variance was explained by function 1, 27.09% explained by function 2, 14.65% explained by function 3, 5.12% explained by function 4 and 2.18% explained by function 5. The Wilks’ lambda values (Table 3) for the functions 1, 2, 3, 4 and 5 were 0.005, 0.039, 0.170, 0.479 and 0.786, respectively, with *p*-values 0.0001, 0.0001, 0.004, 0.199 and 0.486. Table S3 shows the degree of association between chemical composition and the discriminant functions. The first three functions have a high discriminant power. The LDA showed good predictive ability for classification of the geographical origin of Argan oil from the six provinces (Figure 5). The overlap between Taroudant and Tiznit is explained by the geographical similarities previously detected as shown in (Figure 2). 

Discriminant models allowed prediction of the geographical origin of 92.70% of the Argan oil samples (Table 4). Safi, Essaouira and Agadir Ida Outanane presented the highest classification rate (100%), followed by Taroudant (90%), Sidi Ifni (87.5%) and Tiznit (85.7%). According to results obtained by Miklavčič et al. [20] using Orthogonal partial least squares discriminant analysis (OPLS-DA) based on fatty acid profiling, the classification rate varied between 82% and 100%, which was similar to the predictive ability obtained using LDA models (85.7–100%). It also confirms that using a combination of fatty acids and isotope ratios can generate models with high predictive ability. In contrast, Kharbach et al. [11] reported that models built from the chemical composition are not the best for prediction of geographical origin. Nonetheless, samples extracted under different conditions can influence the chemical composition [33]. Therefore, samples extracted under the same conditions are highly recommended to build accurate models with a good predictive ability. LDA is one of the important techniques for dimensionality reduction. However, the LDA has negative aspects such as sensitivity to outliers [34], a linearity problem (equality of averages) [32], and a small sample size which may lead to low robustness and efficiency of one vintage.

## 4. Conclusions

The combination of fatty acid and stable isotope content, coupled with multivariate analyses, such as LDA, was implemented in order to classify Argan oil from six main geographical origins of the natural Argan tree population. The LDA models provided good predictions and were able to discriminate accurately between 92.70% of samples. The combination of fatty acid and stable isotope profiles is a very promising tool for enhancing the Protected Geographical Indication label. Stable isotopes, especially δ^13^C, and fatty acids C16:0, C18:2, unsaturated and saturated fatty acids, showed good relationships with the geographical parameters. Therefore, these can be considered as accurate geographical markers. Further improvement by increasing the number of samples from different production years and considering other purity parameters is recommended for refining and strengthening the models predictive of geographic origin.

## Figures and Tables

**Table 1:** Fatty acid composition of Argan oils from different geographical origins.

	Fatty Acid	Safi (*n* = 3)	Essaouira (*n* = 9)	Agadir Ida Outanane (*n* = 4)	Taroudant (*n* = 10)	Tiznit (*n* = 7)	Sidi Ifni (*n* = 8)	EVAO (SNIMA, 2003)
Pentadecylic acid	C15:0 *	0.06 ± 0.01 ^b^	0.06 ± 0.01 ^ab^	0.05 ± 0.01 ^a^	0.05 ± 0.01 ^ab^	0.05 ± 0.01 ^ab^	0.05 ± 0.01 ^a^	≤0.2%
Palmitic acid	C16:0 ***	13.83 ± 0.64 ^a^	14.50 ± 0.61 ^ab^	13.93 ± 0.05 ^a^	15.85 ± 0.89 ^c^	15.36 ± 0.61 ^bc^	14.98 ± 0.46 ^abc^	11.5–15%
Palmitoleic acid	C16:1 **	0.10 ± 0.01 ^ab^	0.10 ± 0.02 ^ab^	0.09 ± 0.01 ^a^	0.11 ± 0.01 ^ab^	0.10 ± 0.01 ^ab^	0.12 ± 0.01 ^b^	
Margaric acid	C17:0 **	0.09 ± 0.03 ^b^	0.07 ± 0.02 ^b^	0.01 ± 0.01 ^a^	0.06 ± 0.02 ^b^	0.06 ± 0.02 ^b^	0.04 ± 0.03 ^ab^	
Stearic acid	C18:0 *	5.65 ± 0.42 ^a^	5.32 ± 0.60 ^a^	5.39 ± 0.09 ^a^	5.68 ± 0.81 ^a^	6.31 ± 0.45 ^a^	5.69 ± 0.31 ^a^	4.3–7.2%
Oleic acid	C18:1 ω9 *	44.75 ± 0.06 ^a^	46.67 ± 1.98 ^ab^	48.87 ± 0.06 ^b^	48.80 ± 2.06 ^b^	46.17 ± 3.14 ^ab^	46.73 ± 1.74 ^ab^	43.1–49%
Linoleic acid	C18:2 **	35.23 ± 0.02 ^b^	32.84 ± 1.45 ^ab^	31.45 ± 0.06 ^ab^	29.19 ± 2.42 ^a^	31.40 ± 3.35 ^ab^	32.12 ± 1.86 ^ab^	29.3–36%
Linolenic acid	C18:3 ***	0.13 ± 0.04 ^c^	0.06 ± 0.02 ^b^	0.00 ± 0.00 ^a^	0.06 ± 0.03 ^b^	0.09 ± 0.03 ^bc^	0.05 ± 0.04 ^ab^	≤0.3%
Arachidic acid	C20:0 ^NS^	0.09 ± 0.08 ^ab^	0.17 ± 0.10 ^ab^	0.03 ± 0.06 ^a^	0.17 ± 0.11 ^ab^	0.23 ± 0.10 ^b^	0.12 ± 0.10 ^ab^	≤0.5%
Gadoleic acid	C20:1 *	0.1 ± 0.09 ^ab^	0.24 ± 0.11 ^b^	0.04 ± 0.08 ^a^	0.18 ± 0.12 ^ab^	0.21 ± 0.09 ^ab^	0.12 ± 0.08 ^ab^	≤0.5%
	UFA ***	80.32 ± 0.15 ^c^	79.93 ± 0.98 ^bc^	80.45 ± 0.07 ^c^	78.36 ± 1.44 ^ab^	77.98 ± 0.81 ^a^	79.16 ± 0.63 ^abc^	
Saturated fatty acid	SFA ***	19.74 ± 0.18 ^a^	20.13 ± 0.96 ^ab^	19.42 ± 0.11 ^a^	21.83 ± 1.35 ^bc^	22.03 ± 0.66 ^c^	20.90 ± 0.69 ^abc^	

Values are expressed as the mean ± SD. Different letters in the same row designate significant differences (*p* < 0.05). NS = not significant. *** *p* = 0.001, ** *p* = 0.01, * *p* = 0.05. UFA: unsaturated fatty acid; SFA: saturated fatty acid.

**Table 2:** Correlation of stable isotope ratios and fatty acid contents of Argan oil from six provenances with geographical parameters.

	Latitude	Longitude	Altitude	T Max	T Min	Precipitation	Humidity	Distance from Coast
δ^13^C‰	0.01	0.64 ***	0.64 ***	0.26	−0.45 **	0.09	−0.68 ***	0.65 ***
δ^15^N‰	−0.03	−0.10	−0.28	0.07	0.18	−0.10	0.19	−0.12
C15:0	0.49 **	0.13	−0.18	0.08	0.01	0.27	0.12	−0.06
C16:0	−0.38 *	0.43 **	0.41 **	0.54 ***	0.19	−0.49 **	−0.54 ***	0.60 ***
C16:1	−0.38 *	−0.14	−0.07	0.17	0.33 *	−0.47 **	0.02	−0.01
C17:0	0.27	0.15	0.03	0.10	0.05	0.15	0.13	0.09
C18:0	−0.29	0.03	0.04	0.24	0.21	−0.24	−0.13	0.16
C18:1 ω9	−0.09	0.29	0.41**	0.16	−0.25	−0.16	−0.93 *	0.31 *
C18:2	0.28	−0.42 **	−0.52 ***	−0.37 *	0.13	0.35 *	0.56 ***	−0.53 ***
C18:3	0.13	0.01	−0.12	0.08	0.15	0.02	0.07	−0.06
C20:0	−0.04	0.15	−0.03	0.19	0.17	−0.13	−0.26	0.18
C20:1	0.14	0.18	−0.01	0.13	0.10	0.03	−0.16	0.16
UFA	0.43 **	−0.32 *	−0.33 *	−0.45 **	−0.17	0.43 **	0.44 **	−0.52 **
SFA	−0.43 **	0.32 *	0.33 *	0.45 **	0.17	−0.43 **	−0.44 **	0.52 **

Correlation significant at: *** *p =* 0.001, ** *p =* 0.01, * *p =* 0.05. UFA: Unsaturated fatty acid; SFA: saturated fatty acid.

**Table 3:** Discriminant functions elaborated based on the combination of stable isotope ratios and fatty acid composition.

Functions	Wilks’ Lambda	*p*-Value
Function 1 = 6.86 + 1.08 × δ^13^C‰ + 0.15 × δ^15^N‰ + 19.59 × C15:0 + 0.75 × C16:0 − 54.14 × C16:1 − 30.30 × C17:0 − 0.36 × C18:0 + 0.31 × C18:1 + 0.18 × C18:2 − 24.36 × C18:3 + 9.38 × C20:0 − 6.94 × C20:1	0.005	0.000
Function 2 = 14.11 − 0.60 × δ^13^C‰ + 0.77 × δ^15^N‰ + 96.07 × C15:0 − 1.08 × C16:0 − 17.54 × C16:1 + 3.24 × C17:0 + 0.79 × C18:0 − 0.12 × C18:1 − 0.25 × C18:2 + 3.34 × C18:3 − 14.91 × C20:0 + 14.02 × C20:1	0.038	0.000
Function 3 = −101.19 + 0.01 × δ^13^C‰ − 0.01 × δ^15^N‰ + 32.66 × C15:0 + 2.09 × C16:0 − 35.76 × C16:1 + 15.14 × C17:0 + 0.02 × C18:0 + 0.93 × C18:1 + 0.79 × C18:2 + 11.33 × C18:3 − 0.73 × C20:0 + 3.99 × C20:1	0.169	0.004
Function 4 = −48.29 + 0.67 × δ^13^C‰ + 0.24 × δ^15^N‰ + 55.88 × C15:0 − 0.33 × C16:0 + 31.28 × C16:1 − 16.05 × C17:0 + 1.45 × C18:0 − 0.54 × C18:1 + 0.63 × C18:2 + 22.23 × C18:3 − 4.98 × C20:0 − 1.94 × C20:1	0.479	0.199
Function 5 = −153.08 − 0.04 × δ^13^C‰ + 0.29 × δ^15^N‰ − 21.85 × C15:0 + 1.92 × C16:0 − 13.57 × C16:1 + 7.81 × C17:0 + 0.24 × C18:0 + 1.57 × C18:1 + 1.53 × C18:2 − 1.66 × C18:3 − 2.80 × C20:0 + 0.35 × C20:1	0.785	0.486

**Table 4:** Performance of the LDA model for prediction of origin of Argan oil from the six provinces.

Province of Origin	Predicted Origin					
	Agadir Ida Outanane	Essaouira	Safi	Sidi Ifni	Taroudant	Tiznit
Agadir Ida Outanane	4 (100%)	0	0	0	0	0
Essaouira	0	9 (100%)	0	0	0	0
Safi	0	0	3 (100%)	0	0	0
Sidi Ifni	0	0	1 (12.5%)	7 (87.5%)	0	0
Taroudant	0	0	0	0	9 (90%)	1 (10%)
Tiznit	0	0	0	0	1 (14.3%)	6 (85.7%)

## Data Availability

The data presented in this study are contained within the article.

## Supplementary Materials

The following are available online at https://www.mdpi.com/article/10.3390/foods10061274/s1, Table S1: geographical parameters of six sampled provinces of Argan trees. Table S2: isotopes and fatty acid composition of argan oils from six provenances. Table S3: canonical discriminant function coefficients with classification parameters.
