# Supplementary material for: Combination of Stable Isotopes and Fatty Acid Composition for Geographical Origin Discrimination of One Argan Oil Vintage
Source: Foods. 2021 Jun 3;10(6):1274. doi: 10.3390/foods10061274 (PMC8229091; doi:10.3390/foods10061274)
Supplement: Supplementary file 1 [file foods-10-01274-s001.zip › foods-1221806-supplementary.pdf]

Table S1. Geographical parameters of six sampled provenances of Argan trees.

Province	Code	Latitude	Longitude	Altitude (mASL)	Temperature (°C)		Rainfall (mm/year)	Humidity (%)	Distance from Coast (Km)
					Max	Min			
Agadir Ida Outanane	Ai01	30.49	−9.48	449	28.35	12.66	264.40	49.36	18.44
Agadir Ida Outanane	Ai02	30.76	−9.47	431	26.36	13.56	288.85	48.62	34.73
Agadir Ida Outanane	Ai03	30.62	−9.35	769	26.36	13.56	288.85	48.62	41.72
Agadir Ida Outanane	Ai04	30.73	−9.21	782	26.36	13.56	288.85	48.62	61.03
Essaouira	Es01	30.99	−9.61	592	31.01	17.10	236.24	59.78	19.77
Essaouira	Es02	31.10	−9.46	701	31.89	17.26	302.94	51.07	36.61
Essaouira	Es03	31.27	−9.65	624	28.58	16.98	206.60	45.50	14.49
Essaouira	Es04	31.54	−9.35	400	28.84	17.75	296.95	60.31	35.65
Essaouira	Es05	31.64	−9.18	352	29.62	18.19	296.95	60.31	46.66
Essaouira	Es06	31.44	−9.68	104	25.77	17.90	264.08	63.33	10.49
Essaouira	Es07	31.59	−9.10	397	31.30	18.21	296.95	60.31	55.53
Essaouira	Es08	31.44	−9.68	103	23.19	15.66	269.04	65.06	11.28
Essaouira	Es09	31.63	−9.17	356	24.97	14.24	286.04	62.11	48.70
Safi	Sa01	32.03	−9.31	112	29.6	18.44	320.36	70.16	5.57
Safi	Sa02	32.01	−9.32	120	28.96	18.78	320.36	70.16	4.61
Safi	Sa03	31.93	−9.39	124	29.13	18.14	296.95	60.31	3.12
Sidi Ifni	Si01	29.27	−10.08	238	26.09	19.89	155.92	63.52	17.58
Sidi Ifni	Si02	29.16	−10.16	505	31.92	17.99	155.92	63.52	22.27
Sidi Ifni	Si03	29.39	−9.93	349	29.18	19.15	155.92	63.52	21.95
Sidi Ifni	Si04	29.50	−9.93	232	26.81	19.46	205.47	58.39	14.15
Sidi Ifni	Si05	29.37	−9.96	204	26.98	19.84	205.47	58.39	11.16
Sidi Ifni	Si06	29.41	−9.94	347	29.48	19.42	205.47	58.39	14.14
Sidi Ifni	Si07	29.52	−9.89	450	29.25	19.13	205.47	58.39	17.01
Sidi Ifni	Si08	29.14	−10.10	502	23.77	16.18	197.48	65.55	31.48
Taroudant	Ta01	30.35	−8.65	878	36.43	17.59	206.60	45.50	91.15
Taroudant	Ta02	30.41	−8.68	455	35.13	18.04	200.99	39.09	93.21
Taroudant	Ta03	30.44	−8.56	551	34.59	18.84	200.99	39.09	106.86
Taroudant	Ta04	30.56	−8.09	1025	37.20	17.19	200.99	39.09	171.33
Taroudant	Ta05	30.61	−8.08	897	37.20	17.19	201.72	37.99	175.46
Taroudant	Ta06	30.76	−8.41	760	37.93	18.54	201.72	37.99	135.10
Taroudant	Ta07	30.67	−8.88	802	33.95	19.13	201.72	37.99	93.74
Taroudant	Ta08	30.04	−8.31	1297	25.60	10.93	243.67	39.01	133.02
Taroudant	Ta09	30.25	−8.48	1326	25.60	10.93	243.67	39.01	110.85
Taroudant	Ta10	30.40	−8.69	438	26.58	11.91	233.33	41.67	87.43
Tiznit	Ti01	29.53	−9.42	599	34.43	19.61	205.47	58.39	62.42
Tiznit	Ti02	29.66	−9.43	424	32.41	19.94	206.60	45.50	51.47
Tiznit	Ti03	29.69	−9.32	497	32.83	19.60	206.60	45.50	62.54
Tiznit	Ti04	29.71	−9.31	742	34.59	18.50	206.60	45.50	61.04
Tiznit	Ti05	29.65	−9.42	236	32.64	19.72	206.60	45.50	45.29
Tiznit	Ti06	29.80	−9.36	257	32.41	19.94	206.60	45.50	62.57
Tiznit	Ti07	29.57	−9.03	1099	26.15	13.15	253.86	48.48	98.68

mASL: Meters above sea level.

Table S2. Canonical discriminant function coefficients with classification parameters.

	Function				
	**1**	**2**	**3**	4	5
δ13C‰	0.48*	0.59	0.30	0.39	−0.23

C16:1	−0.11	−0.39*	−0.05	0.04	0.31
C18:2	−0.25	0.33*	−0.29	−0.02	−0.17
C15:0	−0.10	0.29*	0.26	0.06	0.08
C16:0	0.09	−0.49	0.57*	0.02	0.01
C17:0	−0.21	0.11	0.44*	0.12	0.02
C18:3	−0.24	0.07	0.31	0.46*	−0.38
δ15N‰	−0.15	−0.09	0.07	0.31*	0.23
C20:0	−0.03	−0.08	0.32	−0.11	−0.57*
C18:0	−0.01	−0.14	0.07	0.23	−0.55*
C20:1	−0.05	0.03	0.38	−0.34	−0.47*
C18:1	0.22	−0.14	0.06	−0.03	0.43*
Eigenvalue	6.35	3.38	1.82	0.64	0.27
Variance %	50.94	27.09	14.65	5.12	2.18
Cumulative %	50.94	78.03	92.69	97.81	100
Canonical correlation	0.93	0.88	0.80	0.62	0.46

*Highest absolute correlation between variable and discriminant function.
